# Genome-Wide Association of Stem Water Soluble Carbohydrates in Bread Wheat

**DOI:** 10.1371/journal.pone.0164293

**Published:** 2016-11-01

**Authors:** Yan Dong, Jindong Liu, Yan Zhang, Hongwei Geng, Awais Rasheed, Yonggui Xiao, Shuanghe Cao, Luping Fu, Jun Yan, Weie Wen, Yong Zhang, Ruilian Jing, Xianchun Xia, Zhonghu He

**Affiliations:** 1 Institute of Crop Science/National Wheat Improvement Center, Chinese Academy of Agricultural Sciences, Beijing, China; 2 College of Agronomy, Xinjiang Agricultural University, 311 Nongda East Road, Urumqi, Xinjiang, 830052, China; 3 International Maize and Wheat Improvement Center (CIMMYT) China Office, Chinese Academy of Agricultural Sciences, Beijing, China; 4 Cotton Research Institute, Chinese Academy of Agricultural Sciences, Anyang, Henan, China; Montana State University Bozeman, UNITED STATES

## Abstract

Water soluble carbohydrates (WSC) in stems play an important role in buffering grain yield in wheat against biotic and abiotic stresses; however, knowledge of genes controlling WSC is very limited. We conducted a genome-wide association study (GWAS) using a high-density 90K SNP array to better understand the genetic basis underlying WSC, and to explore marker-based breeding approaches. WSC was evaluated in an association panel comprising 166 Chinese bread wheat cultivars planted in four environments. Fifty two marker-trait associations (MTAs) distributed across 23 loci were identified for phenotypic best linear unbiased estimates (BLUEs), and 11 MTAs were identified in two or more environments. Liner regression showed a clear dependence of WSC BLUE scores on numbers of favorable (increasing WSC content) and unfavorable alleles (decreasing WSC), indicating that genotypes with higher numbers of favorable or lower numbers of unfavorable alleles had higher WSC content. *In silico* analysis of flanking sequences of trait-associated SNPs revealed eight candidate genes related to WSC content grouped into two categories based on the type of encoding proteins, namely, defense response proteins and proteins triggered by environmental stresses. The identified SNPs and candidate genes related to WSC provide opportunities for breeding higher WSC wheat cultivars.

## Introduction

Bread wheat (*Triticum aestivum* L.) is a widely grown cereal crop globally, feeding nearly one-half of the world population and supplying one-fifth of total food nutrition [1]. It is estimated that global food production in 2050 will be 60% higher than in 2007 [2]. Therefore, it is important to ensure sustainable wheat production for the growing population despite the potentially adverse threats of climate change [3].

Drought and heat stresses, the most important abiotic factors affecting wheat production hinder increases in grain yield. There are many ways to improve resistance to abiotic stresses, including increased wheat stem reserves, improved vigor of root systems and improved photosynthetic efficiency [4–5]. Currently, improvement of the rate of dry matter accumulation is a widely adopted way of making significant progress [5]. Water soluble carbohydrates (WSC) stored in stems and leaf sheaths are important in buffering grain yield potential against hostile environments during the grain filling period [6]. WSC not only contribute to grain growth as the major carbon resource for grain yield, but also contribute in osmotic regulation as the osmolyte [7–8]. Mobilization of WSC during grain filling potentially contributes to 10–20% of final grain weight under normal conditions and up to 30–50% of grain dry matter under drought stress [9–11]. WSC content in wheat stems showed a highly positive relationship with final grain weight, particularly in water-limited environments [12–13]. The grain filling rate, grain weight, and yield in high WSC content cultivars increased by 41, 34 and 10% relative to lower WSC content cultivars, respectively [14]. The release of representative cultivars in Australia and the United Kingdom were associated with increasing WSC content [15], indicating that high stem WSC was a potentially useful trait for improving grain weight and yield [13,16–17].

WSC also fulfil an important role in biotic and abiotic stress conditions. Firstly, various studies indicated that WSC content of cold-tolerant cultivars were higher than in less tolerant cultivars [18]. Secondly, WSC not only supply energy required for plant defense, but also serve as signals for the regulation of defense genes [19–21]. Overall, WSC are involved in a complex communication system necessary for coordination of metabolism with growth, development, and response to environmental changes and stress [22–23].

Although stem WSC accumulation was influenced by many environmental factors [7–8] genomic ranking of wheat cultivars for WSC was consistent across environments, with large broad-sense heritability (*h*^*2*^) of 0.78–0.90 [13,24]. This indicates that variation in WSC content is largely genetically determined [17] and that selection for high WSC should be possible at the early generation stage of a breeding program. Thus, knowledge of the genomic locations, molecular mechanisms and genotypic variation in WSC is critical for understanding yield-limiting factors and for improving yield potential in wheat [24]. During the last decade, QTL for WSC content in wheat were mapped using various types of bi-parental populations, and besides the known major loci, numerous additional chromosomal regions influencing stem WSC were identified [24]. In addition, co-location of QTL for agronomic traits, such as plant height [11] and drought tolerance [25] with QTL for WSC indicated pleiotropic effects of stem WSC. However, linkage mapping has limitations because it only detects favorable alleles present in parental lines.

Association studies (GWAS) based on germplasm collections or specifically designed populations of plants have become a powerful means of dissection of complex quantitative traits and enable identification of loci with novel and superior alleles in diverse populations [26]. Li et al. [27] conducted the first GWAS study of WSC content in 262 cultivars with 209 SSR markers. However, the relatively small numbers of available SSR markers had a limited ability to detect loci controlling WSC content, thus necessitating an improved approach. To date, no GWAS study on WSC content with SNP markers has been published for bread wheat. In this study, we performed a GWAS with a panel of 166 Chinese wheat cultivars using 18,207 mapped SNP markers from the 90K iSelect wheat chip. The aims were to: (1) carry out a genome wide search in bread wheat and identify elite alleles associated with stem WSC content, and (2) search for candidate genes involved in carbohydrate metabolic pathways.

## Materials and Methods

### Plant materials and phenotypic evaluation

One hundred and sixty-six cultivars and advanced lines were used in this study (S1 File), including 144 genotypes from the Yellow and Huai River Valley Facultative Wheat Region of China, nine from Italy, seven from Argentina, four from Japan, and one from Australia, and one from Turkey. They were grown at Anyang (Henan province) and Suixi (Anhui province) during the 2013–2014 cropping season, permitted by the Cotton Research Institute, Chinese Academy of Agricultural Sciences, and at Anyang and Shijiazhuang (Hebei province) during the 2014–2015 cropping season, permitted by the Cotton Research Institute and Institute of Crop Science, Chinese Academy of Agricultural Sciences, providing data for four environments. All cultivars were planted at the beginning of October and harvested in the following mid-June. The field trials were managed as randomized complete blocks with three replicates. Each plot contained three 2 m rows spaced 20 cm apart.

Detailed methods for determination of WSC content were reported previously [28]. For each plot, 20 stems with the same heading date were cut at the soil surface to about 20 cm above the ground at 14 days post-anthesis (DPA). The stem samples from each line were chipped into 3–5 mm length pieces and the WSC content for each sample was determined by near-infrared reflectance spectroscopy (NIRS) following Wang et al. [29]. NIRS regression models employed in this study were highly reliable in determining WSC content as demonstrated by chemical assays of wheat stems (coefficient of determination *R*^*2*^ > 0.992 and root mean square error of prediction RMSEP < 0.228) [29]. Data were collected using the Quant2 package (OPUS 5.0; Bruker Optics). Three independent scans were performed on each sample, and average values were used in subsequent statistical analysis.

### Statistical analysis

Analyses of variance (ANOVA) and correlation coefficients among environments were performed using the SAS System for Windows version 9.0 (SAS Institute, http://www.sas.com). Broad-sense heritability (*h*^*2*^) for WSC content was calculated using the formula: *h*^*2*^ = *σ*_*g*_^*2*^ / (*σ*_*g*_^*2*^ + *σ*_*ge*_^*2*^/*r* +*σ*_*ε*_^*2*^/*re*), where *σ*_*g*_^*2*^, *σ*_*ge*_^*2*^ and *σ*_*ε*_^*2*^ were estimates of genotype (line), genotype × environment interaction and residual error variances, respectively, and *e* and *r* were the numbers of environments and replicates per environment, respectively.

Each year-location combination was treated as an environment. Best linear unbiased evaluation (BLUE) across four environments were calculated using the software package GenStat 14th edition (VSN International, Hemel Hempstead, Hertfordshire, UK) as described in Kollers et al. [30] with genotype and environment as fixed effects; u represents an overall mean and e is a residual term (y = u + genotype + environment + e).

### Genotyping and quality control

Of the 81,587 SNP markers from the wheat 90K SNP iSelect array, 40,267 were mapped to individual chromosomes. Gene diversity, minor allele frequency (MAF) and polymorphism information content (PIC) were calculated by PowerMarker V3.25 [31]. A total of 18,207 scorable, polymorphic markers were employed in our association panel by considering all polymorphic markers with a MAF > 0.05, major allele frequency < 0.5, missing values < 10%, and heterozygosis < 10%. The remaining SNP markers were integrated into a linkage map by inferring marker order and position from the consensus genetic map of the wheat 90K iSelect array [32]. In addition to SNP markers, a gene-specific CAPS marker *WSC7D* for *TaSST-D1* influencing WSC content in wheat was also used to assess allelic and haplotype effects; it generated fragments of 633 and 770 bp in cultivars with *Hap-7D-C* (*TaSST-D1a*) and *Hap-7D-G* (*TaSST-D1b*), respectively, exhibiting a significant difference in WSC content between cultivars with *TaSST-D1a* and those with *TaSST-D1b* [28].

### Population structure

**Fig 1:**
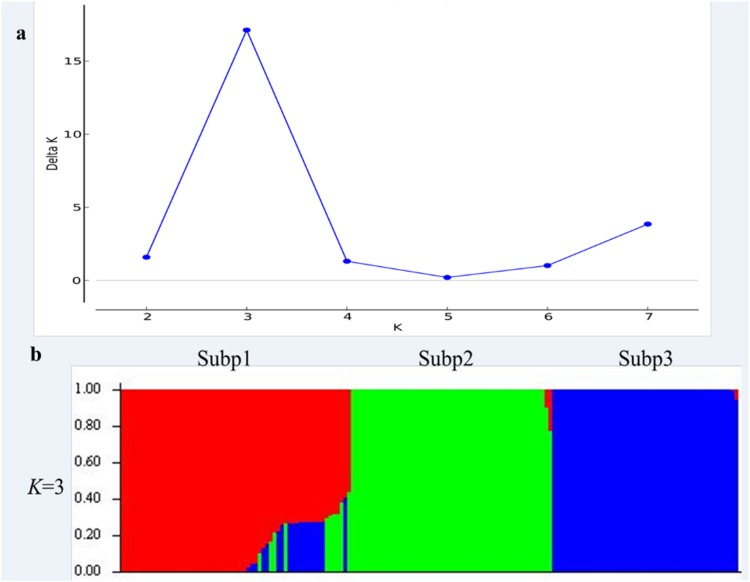
Population structure analysis of 166 cultivars based on unlinked SNP markers. (a) Estimated *Δk* over three repeats of structure analysis; (b) Three sub-populations inferred by structure analysis. Each of the 166 cultivars is represented by a vertical line and different colors indicate different sub-populations.

Population structure was estimated with 5,624 polymorphic SNP markers using Structure software V2.3.4, which implements a model based Bayesian cluster analysis [33]. The number of subpopulations (*K*) was set from 1–10 based on admixture and correlated allele frequencies models. For each *K*, three independent runs were produced. Each run was carried out with 10,000 iteration and a 100,000 burn-in period. The optical value of *K* was determined using the delta-*K* method [34]. Here, *K* = 3 was used, and the whole panel was divided into Subp1, Subp2, and Subp3 (Fig 1).

### Association analysis

BLUEs across four environments for each accession were calculated using GenStat edition V14 as described in Kollers et al. [30]. The BLUEs were then used to fit a mixed linear model (MLM) for association analysis. The MLM with population structure and kinship (K)-matrix were implemented in Tassel V5 software, and 18,207 SNP markers with MAF > 0.05. A threshold *P*-value of 0.001 was used to declare significant QTL for WSC content. Significant markers were visualized in a Manhattan plot drawn in the R Language and Environment for Statistical Computing (R version 3.03; http://www.r-project.org/). Important *P* value distributions (observed *P* values against cumulative *P* values, a negative log_10_ scale) were shown with a quantile-quantile plot drawn in R. Flanking sequences from each trait-associated SNP were used to identify candidate genes or trait-related proteins. The sequences were blast in International Wheat Genome Sequence Consortium (IWGSC: https://urgi.versailles.inra.fr/blast/) database and the resulting sequences were used directly in BLASTx searches in the NCBI database.

### The effect of favorable alleles on WSC content

Every SNP marker has a single base substitution, transition or transversion, hence, each SNP comprises two alleles. Marker alleles with a positive effect leading to higher WSC content will be referred as “favorable alleles”, and those leading to lower WSC content as “unfavorable alleles”. The frequencies of favorable and unfavorable alleles were counted for all cultivars and their allelic effects were determined. Regression analysis between favorable, unfavorable alleles and WSC content were conducted using the line chart function in Microsoft Excel 2011.

## Results

### Phenotypic evaluation

Continuous variation was observed across four environments (S1 Fig). The Spearman correlation coefficients among the four environments ranged from 0.74 to 0.88 (*P* < 0.001). The resulting BLUEs for WSC content across all environments ranged from 6.1 to 19.6% with an average of 15.2%. ANOVA was significant for genotypes, environments and their interaction (Table 1). A very high broad-sense heritability (*h*^*2*^ = 0.93) was obtained across the four environments.

**Table 1:** Analysis of variance of WSC content in wheat accessions of the association panel.

Source of variation	DF	Mean of square	F value
**Genotypes**	165	66.38	14.04***
**Environments**	3	2650.10	560.39***
**Replicates**	8	61.57	13.02***
**Genotype × Environment**	495	6.53	1.38**
**Error**	1300	4.73	

***Significant at *P* < 0.001,

**significant at *P* < 0.01

### Marker coverage and polymorphism in bread wheat

The average marker density for this population was 867 per chromosome. SNP markers integrated into the framework genetic map covered a total genetic distance of 3,700 cM, with an average density of one marker per 0.2 cM. The number of markers per chromosome ranged between 50 (chromosome 4D) and 1,824 (chromosome 1B). However, the marker density for D-genome chromosomes was very low (254.4 per chromosome) compared to the A (1,007.7 per chromosome) and B (1,338.9 per chromosome) chromosomes. PIC values ranged from 0.09 to 0.38 with an average of 0.29 (Table 2).

**Table 2:** Basic statistical analysis of SNP markers in bread wheat.

Chr^a^	*N*	MAF^b^		Diversity		PIC^c^	
		Mean	Range	Mean	Range	Mean	Range
**1A**	1176	0.25	0.05–0.50	0.35	0.10–0.50	0.28	0.09–0.38
**1B**	1824	0.31	0.05–0.50	0.40	0.10–0.50	0.32	0.09–0.38
**1D**	464	0.19	0.05–0.47	0.30	0.10–0.50	0.25	0.09–0.37
**2A**	1050	0.28	0.05–0.50	0.36	0.10–0.50	0.29	0.09–0.38
**2B**	1440	0.26	0.05–0.50	0.35	0.10–0.50	0.29	0.09–0.38
**2D**	553	0.33	0.05–0.50	0.40	0.10–0.50	0.31	0.09–0.38
**3A**	875	0.27	0.05–0.50	0.36	0.10–0.50	0.29	0.09–0.38
**3B**	1193	0.28	0.05–0.50	0.37	0.10–0.50	0.30	0.09–0.38
**3D**	213	0.21	0.05–0.49	0.31	0.10–0.50	0.25	0.09–0.38
**4A**	738	0.26	0.05–0.50	0.36	0.10–0.50	0.29	0.09–0.38
**4B**	720	0.24	0.05–0.50	0.34	0.10–0.50	0.28	0.09–0.38
**4D**	50	0.25	0.05–0.48	0.33	0.10–0.50	0.26	0.10–0.37
**5A**	1008	0.28	0.05–0.50	0.37	0.10–0.50	0.30	0.09–0.38
**5B**	1791	0.30	0.05–0.50	0.39	0.10–0.50	0.31	0.09–0.38
**5D**	166	0.28	0.05–0.50	0.36	0.10–0.50	0.29	0.10–0.38
**6A**	1084	0.25	0.05–0.50	0.34	0.10–0.50	0.28	0.09–0.38
**6B**	1315	0.26	0.05–0.50	0.35	0.10–0.50	0.28	0.09–0.38
**6D**	167	0.25	0.05–0.50	0.35	0.10–0.50	0.28	0.09–0.38
**7A**	1123	0.26	0.05–0.50	0.35	0.10–0.50	0.28	0.09–0.38
**7B**	1089	0.28	0.05–0.50	0.36	0.10–0.50	0.29	0.09–0.38
**7D**	168	0.20	0.05–0.47	0.28	0.10–0.50	0.23	0.09–0.37

^a^ *Chr* Chromosome

^b^ *MAF* Minor allele frequency

^c^ *PIC* Polymorphism information content

### Marker-trait association (MTA) analysis

**Fig 2:**
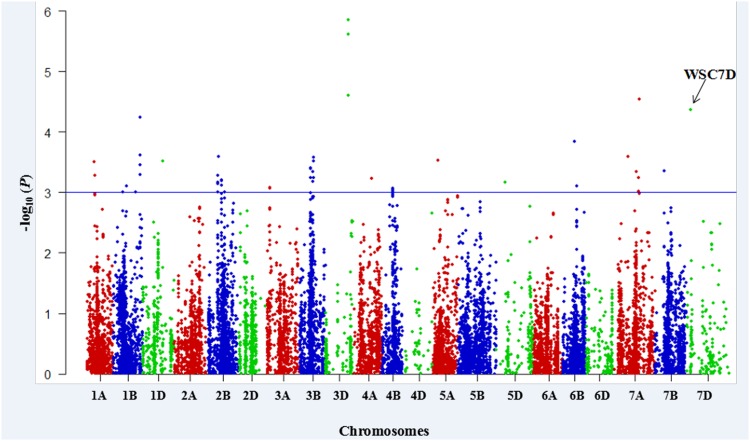
Manhattan plots for statistically significant P values across 21 wheat chromosomes for SNP markers associated with WSC content using the MLM approach. *X*-axis shows SNP markers along each wheat chromosome; *Y-*axis is the -log_10_ (*P*-value), horizontal lines designate 1E-03 threshold for significant associations. The association of gene *TaSST-D1* (WSC7D) with WSC content is shown by black arrows.

**Fig 3:**
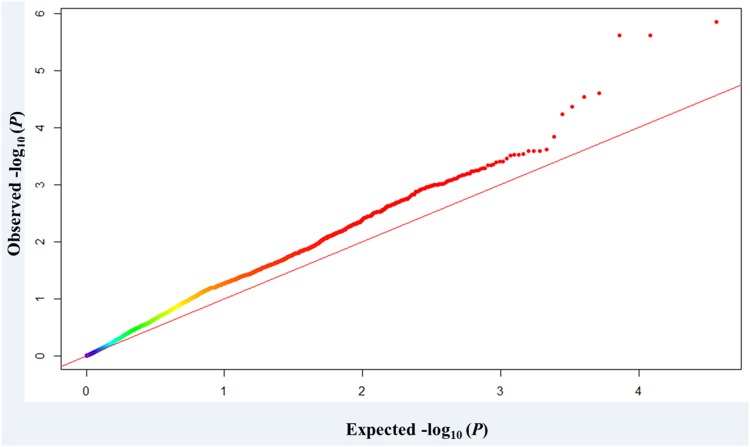
Q-Q plot of SNP associated with WSC using the MLM approach. *X*-axis and *Y*-axis represent cumulative *P*-values and observed *P*-values on a−log_10_ scale, respectively.

The threshold of -log_10_ (*P*-value) ≥ 3.0 (corresponding to a *P-*value < 0.001) was used as a cutoff to identify MTAs. Fifty-two SNPs over 23 loci (significant SNP markers separated by less than 5.0 cM were considered to be the same QTL) were significantly associated with WSC content (Fig 2). Fifty-two MTAs were distributed on all wheat chromosomes except for 2A, 2D, 4D, 5B, 6A and 6D. The maximum number of MTAs were found on chromosomes 2B (9) and 3B (9), followed by 1B (7), while only one MTA was detected on chromosomes 1D, 4A, 5A, 5D, 7B and 7D, respectively. These SNPs represented a MAF ranging from 0.05 to 0.50. The *R*^*2*^ values provided estimates of phenotypic variation explained by MTAs, ranging from 6.8 to 15.2% (Table 3). A quantile-quantile (Q-Q) plot representing expected and observed probability of getting associations of SNPs is presented in Fig 3. The genomic region on chromosome 3D showed a higher peak level significance (*P*-value = 1.41E^-06^, 2.44E^-06^) comprising two SNPs. The known locus WSC7D on chromosome 7DS was also identified in this study (Fig 2; Table 3).

**Table 3:** SNPs significantly associated with WSC content and candidate genes.

Marker^a^	Chr^b^	Pos^c^	Times^d^	SNP^e^	MAF^f^	*P*-value	*R*^*2*^ (%)	Gene^g^	GenBank ID
***wsnp_Ra_c26191_35761997***	1AS	54		**G**/A	0.13	3.14E^-04^	8.2		
***Tdurum_contig8382_300***	1AS	58		**A**/G	0.19	5.25E^-04^	7.6		
***BobWhite_c4147_1429***	1BS	63		A/**G**	0.40	9.91E^-04^	6.8		
***Excalibur_c12994_1060***	1BL	82		**A**/G	0.11	7.83E^-04^	7.7	*RPP8L3*	EMT00042
***Kukri_c11000_1769***	1BL	137		A/**G**	0.19	9.94E^-04^	6.8	*TaMPK21-1*	AKL80629
***BS00066305_51***	1BL	159		G/**A**	0.18	2.42E^-04^	8.6		
***IAAV4884***	1BL	159		**G**/A	0.26	3.51E^-04^	8.1		
***RFL_Contig3165_667***	1BL	159	Two	**A**/G	0.20	5.80E^-05^	10.5		
***BobWhite_c34125_183***	1BL	160		A/**G**	0.25	5.17E^-04^	7.6		
***BS00063907_51***	1DL	116		A/**G**	0.31	3.02E^-04^	8.2	*CBL7*	EMT04707
***Excalibur_c7963_1722***	2BS	69		**A**/G	0.19	5.31E^-04^	7.6	*SDP6*	EMS60550
***GENE-0137_469***	2BS	69		**G**/A	0.19	7.12E^-04^	7.2		
***GENE-1421_706***	2BS	69		**A**/G	0.20	6.75E^-04^	7.6		
***Kukri_c29640_92***	2BS	69		**G**/A	0.18	9.81E^-04^	6.9		
***Kukri_rep_c106290_204***	2BS	69		**G**/A	0.19	7.06E^-04^	7.2		
***Ku_c34562_480***	2BS	72		**A**/G	0.21	2.59E^-04^	8.5	*RPM1*	EMS60551
***Excalibur_c40229_76***	2BS	88		**G**/A	0.27	6.35E^-04^	7.5		
***BS00022949_51***	2BS	91		**G**/A	0.05	7.72E^-04^	7.1		
***BS00065993_51***	2BS	91		**G**/A	0.16	6.31E^-04^	7.5		
***Excalibur_c11505_155***	3AS	26		**A**/G	0.16	8.45E^-04^	7.4	*PPR-repeat*	AGT17134
***RAC875_c20134_535***	3AS	26		**G**/A	0.14	8.22E^-04^	7.2		
***Excalibur_c54388_193***	3B	66	Three	**A**/G	0.09	5.74E^-04^	7.5		
***Kukri_rep_c70097_286***	3B	66	Three	**C**/A	0.08	3.96E^-04^	8.0		
***wsnp_CAP11_c558_382875***	3B	66	Three	**G**/A	0.09	4.00E^-04^	8.0		
***BS00003522_51***	3B	67	Three	**A**/G	0.07	4.11E^-04^	7.9		
***RAC875_c15109_510***	3B	81		A/**G**	0.40	4.53E^-04^	7.9	*Hgsnat*	EMT17170
***TA002089-1495***	3B	81		A/**G**	0.44	6.55E^-04^	7.3		
***RAC875_c35720_229***	3B	82		A/**G**	0.48	5.66E^-04^	7.6		
***RAC875_c35720_456***	3B	82		A/**C**	0.46	3.04E^-04^	8.4		
***wsnp_Ex_rep_c68193_66971396***	3B	83		**G**/A	0.38	2.61E^-04^	8.5		
***BS00067163_51***	3DL	130	Three	**A**/G	0.07	2.44E^-06^	14.5		
***D_GA8KES402JVT1Y_74***	3DL	130	Three	**G**/A	0.07	2.45E^-06^	14.6		
***GENE-1785_118***	3DL	130	Four	**A**/G	0.07	1.41E^-06^	15.2		
***GENE-1785_626***	3DL	130	Three	**A**/G	0.06	2.51E^-05^	12.4		
***Excalibur_c15280_1242***	4AL	109		G/**A**	0.19	5.87E^-04^	7.5		
***BS00062691_51***	4BS	62		C/**A**	0.24	8.45E^-04^	7.0		
***BS00074440_51***	4BS	62		G/**A**	0.25	9.14E^-04^	7.1		
***Tdurum_contig57516_269***	4BS	62		C/**A**	0.25	9.69E^-04^	6.9		
***BS00074439_51***	4BS	63		A/**G**	0.25	9.60E^-04^	7.0		
***GENE-2129_76***	4BS	63		G/**A**	0.24	8.60E^-04^	7.0		
***RAC875_c45747_87***	4BS	63		A/**G**	0.24	8.79E^-04^	7.0		
***RAC875_c33933_350***	5AS	35		G/**A**	0.36	2.92E^-04^	8.4		
***RAC875_rep_c78046_324***	5DL	50		G/**A**	0.18	6.90E^-04^	7.2		
***Excalibur_c58260_332***	6BL	65		**G**/A	0.06	1.43E^-04^	9.2		
***RAC875_c5129_280***	6BL	79		A/**G**	0.26	7.87E^-04^	7.1		
***RAC875_c63889_486***	7AS	88		**A**/G	0.07	2.57E^-04^	8.5	*WAK3*	EMS49185
***wsnp_bq170165A_Ta_1_1***	7AL	136	Two	A/**G**	0.27	4.53E^-04^	8.0		
***tplb0045p11_893***	7AL	148		A/**G**	0.50	5.80E^-04^	7.5		
***IACX2471***	7AL	150		A/**G**	0.44	9.64E^-04^	6.9		
***wsnp_Ku_c42539_50247426***	7AL	152	Three	A/**G**	0.49	2.90E^-05^	11.5		
***RAC875_c26328_75***	7BS	53		**A**/G	0.08	4.42E^-04^	8.0		
***TaSST-D1***	7DS	20		G/**C**	0.41	4.26E^-05^	13.0	*TaSST-D1*	KU376266

^a^ *Marker* Shard markers were detected in MLM models at the threshold -log_10_ (*P*) = 3.0

^b^ *Chr* Chromosome

^c^ *Pos* marker position on the linkage map

^d^ *Times* MTAs identified in number of environments, e.g., two means MTA identified in two environments

^e^ Favorable allele (SNP) is in bold

^f^ *MAF* Minor allele frequency

^g^ *Gene* Candidate gene detected in GenBank

### Relationship between WSC content and numbers of favorable alleles

**Fig 4:**
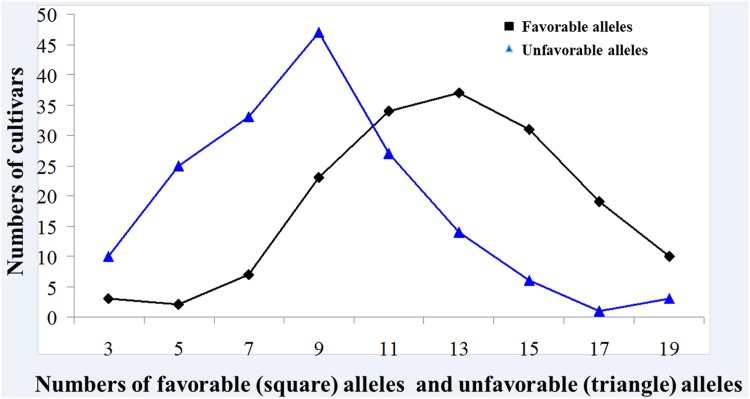
Frequency of favorable and unfavorable WSC alleles in wheat accessions from the association panel.

**Fig 5:**
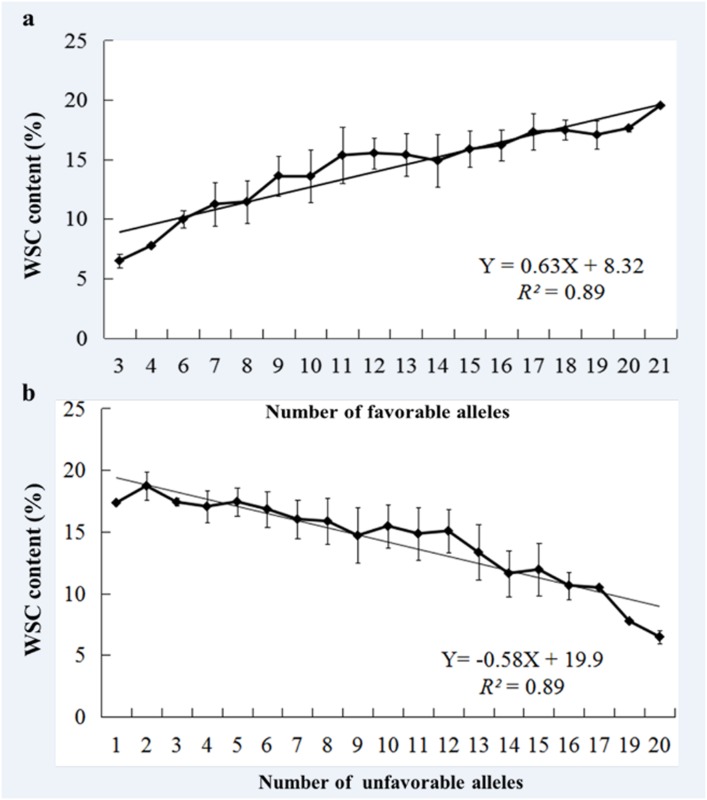
Regression of favorable and unfavorable alleles. Linear regression resulted in a relationship of WSC-BLUEs score and number of favorable and unfavorable alleles in 166 cultivars. The calculations were performed for (a) 23 favorable and (b) 23 unfavorable with significant association with a -log_10_ (*P*-value) ≥3.0.

Individual genotypes contained 0 to 23 favorable alleles (Fig 4). A significant Spearman Rank Order correlation of *r* = 0.95 (*P* < 0.001) was observed between WSC content and number of favorable alleles, with a correlation coefficient *r* = -0.95 (*P* < 0.001) for WSC content and number of unfavorable alleles. Linear regression showed a dependence of the WSC content from the number of favorable alleles with *R*^*2*^ = 0.89 and Y = 0.63 X + 8.32 (Fig 5a); unfavorable alleles were observed with *R*^*2*^ = 0.89 and Y = −0.58 X + 19.9 (Fig 5b). Moreover, combined phenotypic effects were conducted with two selected SNP markers (*BobWhite_c4147_1429* and *Excalibur_c40229_76*) and WSC7D (Table 4). Among these, cultivars such as Aikang 58, Lankao 906, 11CA40, Zhoumai 30, and Neixiang 188 have more favorable alleles and higher WSC content.

**Table 4:** The combined validation for SNP markers (*BobWhite_c4147_1429* and *Excalibur_c40229_76*) and WSC7D.

Combination	Genotype	WSC content (%)	Number (144)	Range
**1**	AAG	11.1	18	6.1–15.3
**2**	AGG	14.6	37	11.2–17.5
**3**	AGC	15.6	22	10.4–18.2
**4**	GAG	16.1	9	12.0–18.3
**5**	AAC	16.2	9	14.1–19.5
**6**	GGG	16.3	23	11.6–19.4
**7**	GAC	17.0	6	14.1–19.6
**8**	GGC	17.3	20	15.2–19.6

The alleles of SNP marker *BobWhite_c4147_1429* was A/**G**, the *Excalibur_c40229_76* was A/**G**, while WSC7D was **C**/G, respectively

### Putative candidate genes associated with significant loci

The blast search gave positive results for 30 flanking sequences of trait-associated SNPs; these represented putative expressed sequences. However, biological functions could be predicted for only 8 sequences. The remaining putatively expressed sequences corresponded to protein sequences without functional annotation. Putative genes associated with significant loci are listed in Tables 3 and 5. Candidate genes were also detected in *Brachypodium distachyon* and *Sorghum*. A few of the candidate genes related to environmental stress; for example, a disease resistance protein and wall-associated receptor kinase 3. The identified candidate genes were roughly divided into two groups according to the types of proteins they encoded (S2 Fig). The first group included genes involved in carbohydrate metabolism such as *TaSST-D1*, *SDP6*, and *Hgsnat*. The second included *CBL7*, PPR-repeat, *RPD8L3*, *RPM1*, *TaMPK21-1*, and *WAK3* associated with stress response.

**Table 5:** Annotation of candidate genes identified by BLASTx.

Gene	Annotation	Reference
***RPP8L3***	*Aegilops tauschii* cultivar AL8/78 disease resistance RPP8-like protein 3	Jia et al. [54]
***TaMPK21-1***	*Triticum aestivum* cultivar Norstar mitogen activated protein kinase 21–1	
***CBL7***	*Aegilops tauschii* cultivar AL8/78 calcineurin B-like protein 7	Jia et al. [54]
***SDP6***	*Triticum urartu* cultivar G1812 glycerol-3-phosphate dehydrogenase SDP6	Ling et al. [55]
***RPM1***	*Triticum urartu* cultivar G1812 disease resistance protein RPM1	Ling et al. [55]
***PPR-repeat***	*Saccharum hybrid* cultivar R570 pentatricopeptide repeat protein	Setta et al. [56]
***Hgsnat***	*Aegilops tauschii* cultivar AL8/78 heparan-alpha-glucosaminide N-acetyltransferase	Jia et al. [54]
***WAK3***	*Triticum urartu* cultivar G1812 wall-associated receptor kinase 3	Ling et al. [55]
***TaSST-D1***	*Triticum aestivum* sucrose: sucrose 1-fructosyltransferase	Dong et al. [28]

## Discussion

### Comparison of Chinese and foreign wheat cultivars

The wheat cultivars used in the present study includes 144 Chinese cultivars and 22 foreign wheats. The population structure analysis indicated that 20 foreign wheat cultivars were classified into Subp1, indicating a similar genetic basis and close relationship with those from Shandong province. In terms of *TaSST-D1* gene associated with stem WSC content, 18 foreign cultivars carried *TaSST-D1b* allele, three had *TaSST-D1a*, and one was heterozygote. In addition, the averaged favorable alleles for foreign cultivars were 10, with a range from 3 to 15, whereas the means of favorable alleles was 14 in Chinese wheat cultivars, ranging from 6 to 21.

### Marker-trait associations for WSC content

Here, we report a GWAS approach for identifying genomic regions associated with WSC content genotyped in a collection of 166 cultivars using 18,207 SNP markers. Previously, GWAS for WSC content was analyzed using low-density SSR markers [27], but this is the first study of GWAS using high-density SNP markers. Hence, the loci identified in the study are difficult to align and compare with the QTL reported by Li et al. [27]. Many QTL related to this trait were previously identified by linkage mapping, and comparison of those QTL to our studies may help to validate the importance of these loci in enhancing WSC content.

Yang et al. [35] identified 20 QTL related to WSC at the flowering, grain filling and maturity stages using a doubled haploid mapping population. They found that *QAeswc*.*cgb-1A*.*1*, *QAeswc*.*cgb-2A*.*1*, *QAeswc*.*cgb-5A*, and *QAeswc*.*cgb-7B* were involved in very significant interactions with drought stress. In our study, MTAs were detected on chromosomes 1A, 5A, and 7B, suggesting the importance of exploring the relationship between these loci and drought stress. Rebetzke et al. [11] identified 33 QTL related to WSC content distributed among 21 chromosomal regions. A QTL on 4BS mapped near the gibberellin-insensitive dwarfing gene *Rht-B1*. We identified one locus comprising six SNPs on chromosome 4BS, indicating that some functional genes within this region influencing WSC content were likely to be linked with *Rht-B1*. Zhang et al. [24] identified 49 loci for WSC at 20 chromosome locations, among which markers on chromosomes 3B, 3D, 5D and 7B made positive contributions to thousand grain weight (TGW) under well-watered, drought and heat stress conditions. Two haplotypes of four and five SNPs on chromosome 3B detected in the current study were located in the proximity of previously mapped QTL. Similarly, a haplotype block of four SNPs on chromosome 3DL should be further investigated for a role in drought tolerance. Li et al. [27] used GWAS to map WSC loci in 262 winter wheat lines with 209 SSR markers and identified 16 QTL distributed over 11 chromosomes. Among these, chromosomes 1B, 2B, 2D, 4B, and 5D contributed to significantly higher TGW. We identified one haplotype of four SNPs on chromosome 1BL and another haplotype of six SNPs on 2BS significantly associated with WSC content. This indicated that WSC played an important role in environmental stress and SNP markers in these regions should enable selection of cultivars with higher WSC. In addition, many studies demonstrated that chromosome 5D carried important stress response genes, conferring salt and drought tolerance [36,37]. Akpinar et al. [38] sequenced chromosome 5D of *Aegilops tauschii*. In the present study, we detected a MTA at the position of 50 cM on chromosome 5DL. Twelve SNPs between 45 and 59 cM were selected to compare with Akpinar et al. [38]. The flanking sequences of these SNPs were also used to blast against the CDS sequences of *Brachypodium*, rice and *sorghum*. As a result, 8 SNPs got best blast hits in the three species, which were subsequently used to search the relative contigs mentioned in Akpinar et al. [38]. Interestingly, the SNP marker *RAC875_rep_c72023_267* and contig IH6Q7OR01B69G8 have the same blast hit *Bradi4g30270*.*1*, and *wsnp*_*Ex_c9822_16203685* and contig 04556 have the same blast hits *Bradi4g30200*.*1* and *Sb02g024620*.*1*. Moreover, *RAC875_rep_c72023_267* was at a similar position with contig 04556 according to the virtual gene order in chromosome 5D of *Aegilops tauschii* and wheat 90K consensus map. It is necessary to validate the relationship between this SNP and stress tolerance.

### The relationship between loci controlling WSC content and TGW

Various studies reported significant correlations between WSC content and TGW, and a high correlation was detected in our study (*r* = 0.58, *P* < 0.001). Yang et al. [35] reported QTL for stem WSC content, accumulation efficiency, and transportation efficiency sharing some chromosome segments with QTL controlling TGW and grain filling efficiency. On chromosome 2D in particular, QTL for TGW at the period of maturity and stem WSC content at the flowering stage were linked to SSR marker *WMC41*. Similarly, QTL controlling of stem WSC content, WSC accumulation efficiency, and TGW were distributed in the *Xgwm299*—*Xgwm247* interval on chromosome 3B [35]. On chromosome 4A, QTL for stem WSC content and TGW were present in marker intervals of 44.7 cM (*P3446-205*—*P3613-190*) and 10.9 cM (*P5611-136*—*P2454-270*) [35]. The MTAs identified in this study were mainly distributed on chromosomes 1AS, 1BS, 1BL, 1DL, 2BS, 3AS, 3B, 3DL, 4AL, 4BS, 5AS, 5DL, 6BL, 7AS, 7AL, 7BS and 7DS. Interestingly, QTL for grain weight were also detected in these chromosomes. Our previous study mapped three QTL, of which those on chromosomes 4BS and 7AS were associated with both stem WSC content and TGW, indicating that the same chromosomal regions were involved in controlling both traits, and that it is possible to obtain high TGW cultivars by selection for WSC content.

### *In silico* putative candidate gene analysis

WSC act as a complex communication system necessary for coordination of metabolism with growth, development and responses to environmental changes and stresses [22–23]. Previous studies reported that WSC metabolic genes are involved in the Calvin cycle, gluconeogenic, fructan and glycolytic sucrose synthetic pathway, and major carbohydrate metabolic pathways [13]. However, WSC are not only involved in grain growth and development as the main carbon source for grain weight, but also act as an osmolyte in osmotic regulation under diverse environmental conditions [8, 39–42]. Due to the highly repetitive nature of the hexaploid wheat genome and complicated quantitative basis of WSC-related traits, few putative genes controlling WSC content were reported in wheat.

In the present study, eight candidate genes related to WSC content were identified and divided into two groups based on the types of proteins they encoded. Group 1 encoded carbohydrate catabolism proteins. For example, the *SDP6* gene participates in a mitochondrial glycerol-3-P (G3P) shuttle and is essential for glycerol metabolism.

Quettier et al. [43] indicated that mutant alleles of *SDP6* were able to break down triacylglycerol but failed to accumulate soluble sugars. Group 2 candidate genes are probably involved in biotic (disease) and abiotic (wounding, salt, drought and heat) stresses. For example, disease resistance genes *RPP8L3* and *RPM1* were significantly associated with WSC content. WSC is involved in plant immunity because it provides energy for defense response by regulating source/sink relationships and up-regulation of defense gene expression [19]. Secondly, mitogen-activated protein kinase encoded by *TaMPK21-1* reversibly phosphorylates kinases to activate defense gene expression [44]. MPK genes were reported to participate in response to cold, drought, ultraviolet light, oxidation stress and disease in many crops [45–47]. Thirdly, *CBL7*, as one of the plant calcium sensors, can interact with CIPKs to form CBL-CIPK complexes that mediate responses to salinity, drought stress, phosphorous deficiency and ABA signaling [48–50]. Li et al. [49] indicated that over-expression of soybean *CBL1* enhances tolerance to salinity and drought stress in *Arabidopsis*. In addition, the WAK gene plays critical roles in cell expansion, pathogen resistance, and heavy-metal stress tolerance in *Arabidopsis* [51]. Hurni et al. [52] isolated northern corn leaf blight resistance gene *Htn1* that encodes WAK in maize. These candidate genes provide a basis for dissecting the genetic mechanism of WSC and will be useful in further investigations of the various functions of WSC in wheat.

### Potential application of MTAs for MAS in wheat breeding

Increased grain weight in wheat was attributed to significant improvement in stem WSC content [15,53]. Li et al. [27] demonstrated that the average number of favorable WSC alleles increased from 1.13 in pre-1960 varieties period to 4.41 in post-2000 varieties. Thus, characterization of favored loci will assist in selecting parents for wheat breeding programs, in order to ensure maximum numbers of favored loci for selection using SNP markers. In the present study, 52 SNP were detected and the *R*^*2*^ ranged from 6.8 to 15.2%. Similarly, a significant and positive correlation was detected between WSC content and number of favorable alleles (*r* = 0.68, *P* < 0.001). This means that cultivars with relatively higher numbers of favorable alleles, or reduced numbers of unfavorable alleles, will have higher WSC and pyramiding of favorable alleles can be an effective way to improve WSC content in breeding programs. In order to select SNP markers that clearly discriminate two alleles (one allele was associated with higher WSC content, and the other associated with lower WSC), 52 MTAs were separately used to validate the relationships of contrasting alleles with WSC content. Two SNP markers, *BobWhite_c4147_1429* and *Excalibur_c12994_1060* were significantly associated with WSC content. The average WSC contents of the two alleles of *BobWhite_c4147_1429* were 14.2 (genotype AA) and 16.5% (genotype GG), respectively. Similarly, the average WSC of the alleles of *Excalibur_c12994_1060* were 15.6 (genotype AA) and 12.0% (genotype GG), respectively. A validation experiment of combining these SNP markers and the CAPS marker WSC7D developed by Dong et al. [28] was undertaken. Among the eight combinations, those with all three unfavorable alleles had the lowest average WSC content of 11.1% (range 6.1 to 15.3%), whereas the combination with all three favorable alleles had the highest WSC content of 17.3% (range 15.2 to 19.6%). It will be most desirable if these three SNP markers can be transformed into Kompetitive Allele-Specific PCR (KASP) markers for use in marker assisted gene pyramiding in breeding programs.

## Supporting Information

S1 Fig Frequency distribution of WSC content in the 166 cultivar germplasm set. A, Anyang 2013; B, Suixi 2013; C, Anyang 2014; D, Shijiazhuang 2014.
(TIF)

S2 Fig Phylogenetic analysis of candidate genes identified by *in silico* analysis. (TIF)

S1 File The 166 accessions and their origins. (PDF)
