# Supplementary figures and images for: Genome-Wide Association of Stem Water Soluble Carbohydrates in Bread Wheat
Source: PLoS One. 2016 Nov 1;11(11):e0164293. doi: 10.1371/journal.pone.0164293 (PMC5089554; doi:10.1371/journal.pone.0164293)

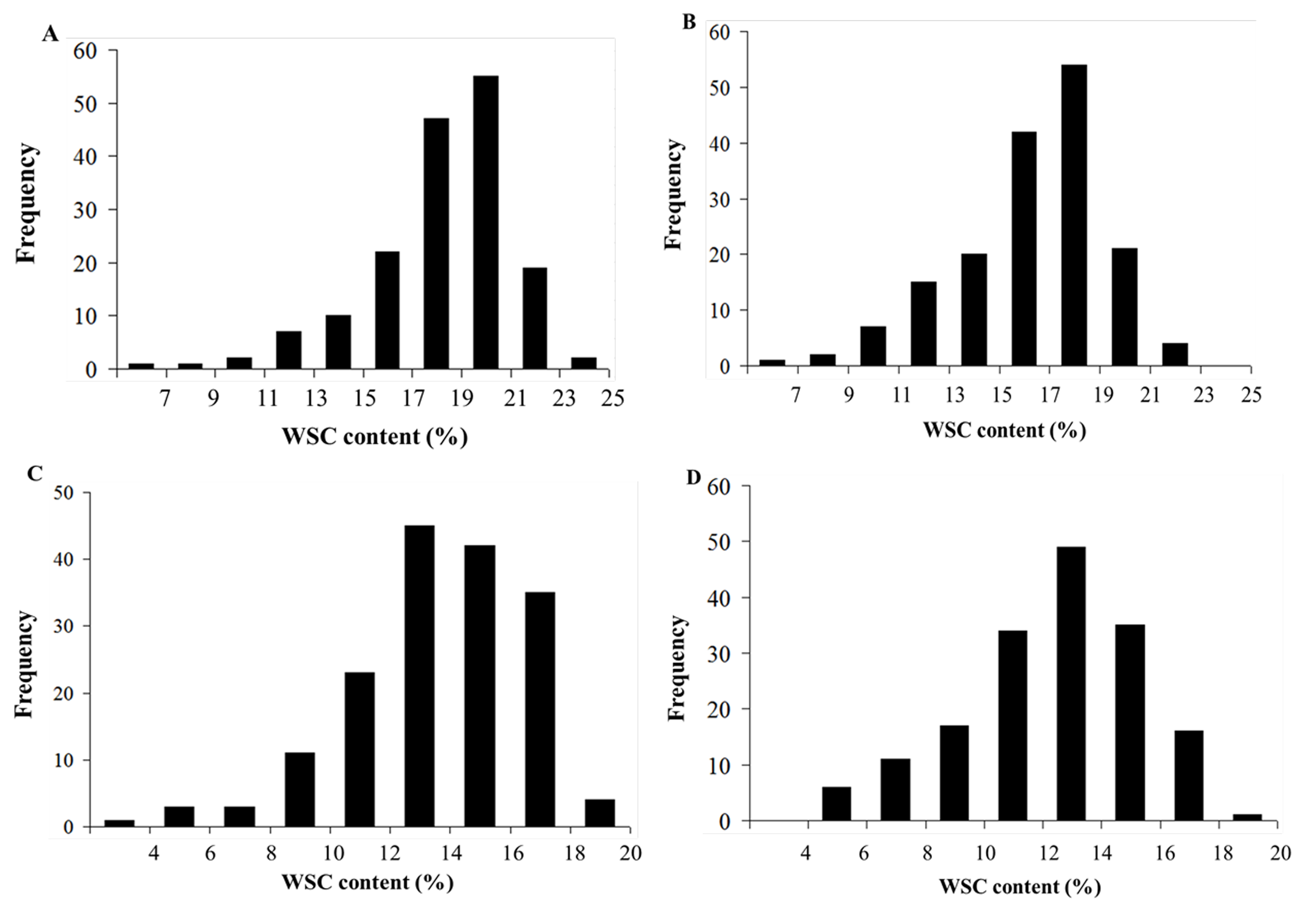

Supplement: S1 Fig — A, Anyang 2013; B, Suixi 2013; C, Anyang 2014; D, Shijiazhuang 2014. (TIF) [file pone.0164293.s001.tif]

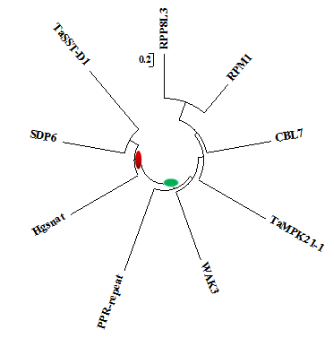

Supplement: S2 Fig — (TIF) [file pone.0164293.s002.tif]
