# Supplementary material for: Genome-Wide Association of Stem Water Soluble Carbohydrates in Bread Wheat
Source: PLoS One. 2016 Nov 1;11(11):e0164293. doi: 10.1371/journal.pone.0164293 (PMC5089554; doi:10.1371/journal.pone.0164293)
Supplement: S1 File — (PDF) [file pone.0164293.s003.pdf]

Table S1 The 166 accessions and their origins

No.	Name	Origin	Number of favorable alleles[a]	BLUE[b]	*TaSST-D1*[c]	Subp[d]
1	An 1331	Anhui	11	17.26	*TaSST-D1b*	2
2	Fu 936	Anhui	11	14.87	*TaSST-D1b*	2
3	Huaimai 18	Anhui	19	15.16	*TaSST-D1a*	1
4	Huaimai 20	Anhui	19	18.34	*TaSST-D1a*	1
5	Huaimai 21	Anhui	14	15.75	*TaSST-D1a*	3
6	Su 0663	Anhui	12	16.33	*TaSST-D1b*	2
7	Sunong 6	Anhui	16	16.73	*TaSST-D1a*	2
8	Wan 23094	Anhui	9	13.43	*TaSST-D1b*	2
9	Wanmai 19	Anhui	10	15.69	*TaSST-D1b*	2
10	Wanmai 29	Anhui	13	14.08	*TaSST-D1b*	2
11	Wanmai 33	Anhui	12	15.67	*TaSST-D1b*	2
12	Wanmai 38	Anhui	14	14.94	*TaSST-D1a*	1
13	Wanmai 50	Anhui	12	17.27	*TaSST-D1b*	2
14	Wanmai 52	Anhui	13	16.11	*TaSST-D1b*	2
15	Wanmai 53	Anhui	11	16.09	*TaSST-D1b*	2
16	Gaocheng 8901	Hebei	7	11.39	*TaSST-D1b*	1
17	Gaoyou 503	Hebei	10	13.06	H	2
18	Hengguan 35	Hebei	17	18.76	*TaSST-D1a*	3
19	Han 6172	Hebei	16	16.50	H	3
20	Heng 7228	Hebei	10	12.58	*TaSST-D1b*	3
21	Hengguan 33	Hebei	14	16.51	*TaSST-D1a*	3
22	Jinhe 9123	Hebei	16	16.77	*TaSST-D1a*	3
23	Jishi 02-1	Hebei	9	11.77	*TaSST-D1b*	2
24	Shi 4185	Hebei	18	16.33	*TaSST-D1a*	3
25	Shijiazhuang 15	Hebei	16	18.37	*TaSST-D1a*	3
26	Shijiazhuang 8	Hebei	12	16.09	*TaSST-D1a*	3
27	Shixin 733	Hebei	15	15.66	*TaSST-D1b*	2
28	Shixin 828	Hebei	15	16.75	*TaSST-D1b*	2
29	Shiyou 17	Hebei	8	13.31	*TaSST-D1b*	1
30	11CA40	Henan	21	19.52	*TaSST-D1a*	1
31	85Zhong 33	Henan	15	14.63	*TaSST-D1b*	3
32	Aikang 58	Henan	17	18.99	*TaSST-D1b*	3
33	Bainong 3217	Henan	15	14.33	*TaSST-D1b*	2
34	Bainong 64	Henan	15	18.19	*TaSST-D1a*	2
35	Huapei 5	Henan	14	18.26	*TaSST-D1b*	3
36	Lankao 2	Henan	17	16.88	*TaSST-D1b*	3

37	Lankao 24	Henan	14	18.34	*TaSST-D1b*	3
38	Lankao 906	Henan	17	19.41	*TaSST-D1b*	3
39	Luohan 2	Henan	13	14.58	*TaSST-D1b*	2
40	Luomai 21	Henan	13	18.14	*TaSST-D1b*	3
41	Neixiang 188	Henan	17	19.64	*TaSST-D1a*	2
42	Neixiang 5	Henan	10	10.21	H	3
43	St1472/506	Henan	13	11.87	*TaSST-D1b*	2
44	Xinmai 19	Henan	13	14.59	*TaSST-D1a*	2
45	Xinmai 9	Henan	12	15.10	*TaSST-D1a*	2
46	Xinmai 9408	Henan	12	15.23	*TaSST-D1b*	2
47	Yanzhan 4110	Henan	15	15.59	*TaSST-D1b*	2
48	Yumai 13	Henan	18	18.06	*TaSST-D1a*	1
49	Yumai 18	Henan	17	17.24	*TaSST-D1a*	2
50	Yumai 2	Henan	10	14.14	*TaSST-D1a*	3
51	Yumai 21	Henan	18	18.17	*TaSST-D1a*	1
52	Yumai 34	Henan	15	17.92	*TaSST-D1a*	1
53	Yumai 35	Henan	17	18.08	*TaSST-D1a*	3
54	Yumai 47	Henan	15	16.83	*TaSST-D1a*	2
55	Yumai 49	Henan	12	17.50	*TaSST-D1a*	2
56	Yumai 50	Henan	16	15.82	*TaSST-D1a*	3
57	Yumai 57	Henan	11	18.22	*TaSST-D1a*	2
58	Yumai 63	Henan	17	15.70	*TaSST-D1b*	2
59	Yumai 7	Henan	20	17.90	*TaSST-D1a*	3
60	Zheng 9023	Henan	18	17.44	*TaSST-D1a*	2
61	Zhengmai 366	Henan	13	15.75	H	2
62	Zhengzhou 3	Henan	8	11.32	*TaSST-D1b*	2
63	Zhong 892	Henan	15	15.73	*TaSST-D1b*	3
64	Zhongmai 871	Henan	10	15.96	*TaSST-D1b*	3
65	Zhongmai 875	Henan	15	18.30	*TaSST-D1a*	3
66	Zhongmai 895	Henan	13	18.34	H	3
67	Zhongyu 5	Henan	16	17.61	*TaSST-D1a*	3
68	Zhongyu 9	Henan	11	16.90	*TaSST-D1a*	3
69	Zhou8425B	Henan	10	16.30	*TaSST-D1b*	3
70	Zhoumai 11	Henan	15	18.20	*TaSST-D1b*	3
71	Zhoumai 12	Henan	10	16.55	*TaSST-D1b*	3
72	Zhoumai 13	Henan	14	15.89	H	3
73	Zhoumai 16	Henan	11	16.78	*TaSST-D1b*	3
74	Zhoumai 18	Henan	13	17.25	*TaSST-D1b*	3
75	Zhoumai 19	Henan	12	16.20	*TaSST-D1b*	2
76	Zhoumai 22	Henan	10	15.83	*TaSST-D1b*	3

77	Zhoumai 23	Henan	13	17.73	H	2
78	Zhoumai 25	Henan	11	18.36	*TaSST-D1a*	3
79	Zhoumai 26	Henan	12	16.57	H	3
80	Zhoumai 28	Henan	11	16.59	*TaSST-D1b*	3
81	Zhoumai 30	Henan	15	19.58	*TaSST-D1a*	3
82	Zhoumai 31	Henan	12	17.51	*TaSST-D1b*	2
83	Zhoumai 32	Henan	14	17.46	*TaSST-D1b*	3
84	Aifeng 3	Shaanxi	11	9.90	*TaSST-D1b*	1
85	Bima 1	Shaanxi	7	13.03	*TaSST-D1b*	1
86	Bima 4	Shaanxi	6	9.47	*TaSST-D1b*	1
87	Fengchan 3	Shaanxi	15	13.11	*TaSST-D1a*	2
88	Shan 150	Shaanxi	15	14.24	H	2
89	Shan 229	Shaanxi	12	14.43	*TaSST-D1b*	2
90	Shan 253	Shaanxi	15	14.49	*TaSST-D1b*	2
91	Shan 354	Shaanxi	14	16.03	*TaSST-D1a*	3
92	Shan 512	Shaanxi	15	14.91	*TaSST-D1a*	2
93	Shan 715	Shaanxi	13	14.33	*TaSST-D1b*	3
94	Shanmai 509	Shaanxi	13	15.78	*TaSST-D1a*	3
95	Shanmai 94	Shaanxi	11	17.16	*TaSST-D1b*	2
96	Shannong 7859	Shaanxi	12	13.79	*TaSST-D1b*	3
97	Shannong 981	Shaanxi	20	17.34	H	2
98	Shanyou 225	Shaanxi	14	15.29	*TaSST-D1a*	2
99	Wunong 148	Shaanxi	13	15.28	*TaSST-D1a*	2
100	Xiaoyan 22	Shaanxi	11	14.47	H	3
101	Xiaoyan 54	Shaanxi	12	15.90	*TaSST-D1a*	2
102	Xiaoyan 6	Shaanxi	10	12.72	H	2
103	Xiaoyan 81	Shaanxi	14	14.65	*TaSST-D1a*	2
104	Xinong 1376	Shaanxi	15	17.33	*TaSST-D1a*	3
105	Xinong 2000-7	Shaanxi	13	15.93	*TaSST-D1a*	2
106	Xinong 291	Shaanxi	14	11.57	*TaSST-D1b*	1
107	Xinong 88	Shaanxi	12	15.47	*TaSST-D1b*	2
108	Xinong 979-005	Shaanxi	9	14.14	*TaSST-D1a*	2
109	Jimai 19	Shandong	13	15.00	*TaSST-D1a*	1
110	Jimai 20	Shandong	9	15.74	H	1
111	Jimai 21	Shandong	17	15.77	*TaSST-D1a*	1
112	Jimai 22	Shandong	19	16.88	*TaSST-D1a*	1
113	Jinan 13	Shandong	15	13.39	*TaSST-D1a*	1
114	Jinan 17	Shandong	14	13.12	*TaSST-D1b*	1
115	Jining 16	Shandong	14	16.76	*TaSST-D1b*	1
116	Liangxing 66	Shandong	18	16.53	*TaSST-D1a*	1

117	Liangxing 99	Shandong	17	15.43	*TaSST-D1a*	1
118	Linmai 2	Shandong	18	18.18	*TaSST-D1b*	1
119	Linmai 4	Shandong	17	17.11	*TaSST-D1b*	1
120	Luami 15	Shandong	12	15.25	*TaSST-D1b*	3
121	Lumai 11	Shandong	15	13.33	*TaSST-D1b*	1
122	Lumai 14	Shandong	19	17.18	*TaSST-D1a*	1
123	Lumai 21	Shandong	11	14.87	*TaSST-D1b*	1
124	Lumai 23	Shandong	19	18.16	*TaSST-D1b*	1
125	Lumai 5	Shandong	12	14.31	H	1
126	Lumai 6	Shandong	16	14.09	*TaSST-D1b*	2
127	Lumai 7	Shandong	9	15.77	*TaSST-D1a*	3
128	Lumai 8	Shandong	17	15.74	*TaSST-D1b*	1
129	Lumai 9	Shandong	15	15.32	*TaSST-D1b*	1
130	Luyuan 502	Shandong	16	15.31	*TaSST-D1b*	1
131	PH82-2	Shandong	11	14.93	H	2
132	Shannong 20	Shandong	17	16.31	*TaSST-D1a*	1
133	Taishan 1	Shandong	10	15.14	H	1
134	Taishan 5	Shandong	13	13.20	*TaSST-D1b*	1
135	Wennong 14	Shandong	19	16.70	*TaSST-D1a*	1
136	Wennong 5	Shandong	11	14.55	*TaSST-D1b*	1
137	Yannong 15	Shandong	9	12.69	*TaSST-D1b*	1
138	Yannong 18	Shandong	16	15.74	*TaSST-D1b*	3
139	Yannong 19	Shandong	16	14.96	*TaSST-D1a*	1
140	Zimai 12	Shandong	14	16.27	*TaSST-D1b*	1
141	Zixuan 2	Shandong	10	13.05	*TaSST-D1b*	1
142	Jinmai 61	Shanxi	20	17.62	*TaSST-D1a*	1
143	Linhan 2	Shanxi	14	14.62	*TaSST-D1a*	2
144	Linkang 12	Shanxi	14	11.98	*TaSST-D1b*	3
	Foreign cultivars					
145	Aca 601	Argentina	8	9.68	*TaSST-D1b*	1
146	Aca 801	Argentina	10	10.61	*TaSST-D1b*	1
147	KleinFlecha	Argentina	14	12.73	*TaSST-D1b*	1
148	KleinJabal1	Argentina	11	11.23	*TaSST-D1b*	1
149	NideraBaguette 10	Argentina	4	7.75	*TaSST-D1b*	1
150	NideraBaguette 20	Argentina	3	6.10	*TaSST-D1b*	1
151	ProINTAColibr 1	Argentina	3	6.87	*TaSST-D1b*	1
152	Sunstate	Austrilia	10	13.59	*TaSST-D1b*	1
153	Abbondanza	Italy	7	11.16	*TaSST-D1b*	1
154	Barra	Italy	6	10.47	*TaSST-D1b*	1
155	Dorico	Italy	9	11.86	*TaSST-D1b*	1

156	Funo	Italy	14	13.45	*TaSST-D1a*	2
157	Genio	Italy	10	9.94	*TaSST-D1b*	1
158	Lampo	Italy	12	13.28	H	1
159	Libero	Italy	7	9.38	*TaSST-D1b*	1
160	Mantol	Italy	13	13.71	*TaSST-D1b*	1
161	Sagittario	Italy	14	13.70	*TaSST-D1b*	1
162	Kanto 107	Japan	15	15.19	*TaSST-D1b*	1
163	Kitanokaori	Japan	10	11.90	*TaSST-D1a*	3
164	Norin 61	Japan	11	13.36	*TaSST-D1b*	1
165	Norin 67	Japan	12	13.60	*TaSST-D1b*	1
166	HK1/6/NVSR3/5/BEZ/TVR/5/CFN/BEZ//SU92/CI13645/3NAI60	Turkey	14	10.39	*TaSST-D1a*	1

[a] Number of favorable alleles detected

[b] BLUE of WSC content across four environments.

[c] Genotype at the *TaSST-D1* locus, H indicates heterogeneity at this locus.

[d] 166 cultivars were grouped into three sub-populations, 1, 2, 3 indicate sub-populations
